# A novel perspective of associativity of upper limb motor impairment and cortical excitability in sub-acute and chronic stroke

**DOI:** 10.3389/fnins.2022.832121

**Published:** 2022-07-25

**Authors:** Megha Saini, Neha Singh, Nand Kumar, M. V. Padma Srivastava, Amit Mehndiratta

**Affiliations:** ^1^Centre for Biomedical Engineering, Indian Institute of Technology Delhi, New Delhi, India; ^2^Department of Psychiatry, All India Institute of Medical Sciences, New Delhi, India; ^3^Department of Neurology, All India Institute of Medical Sciences, New Delhi, India; ^4^Department of Biomedical Engineering, All India Institute of Medical Sciences, New Delhi, India

**Keywords:** stroke, Fugl-Meyer scale, motor evoked potential, neurophysiological parameters, Transcranial Magnetic Stimulation, corticospinal tract, Resting Motor Threshold

## Abstract

**Background:**

The global inclination of stroke onset in earlier years of life and increased lifespan have resulted in an increased chronic post-stroke-related disability. The precise and simplistic approach such as the correlation of Fugl-Meyer Assessment (FMA) with Transcranial Magnetic Stimulation (TMS) parameters, Resting Motor Threshold (RMT) and Motor Evoked Potential (MEP), in patients with stroke might play a critical role, given the prognostic value of MEP, a measure of cortical excitability, and might be the key point in prescribing appropriate therapeutic strategies.

**Objective:**

The study aimed to determine the correlation of FMA-based impairment in the upper extremity function specifically of the wrist and hand with respect to the neurophysiological parameters of corticospinal tract integrity.

**Materials and methods:**

The Institutional Review Board approved the study and 67 (n) patients with stroke were enrolled in the Department of Neurology, AIIMS, New Delhi, India. The motor assessment was performed on patients by the upper extremity subset of Fugl-Meyer Assessment (FMA) and the clinical history was obtained. RMT and MEP of Extensor Digitorum Communis (EDC) muscle were measured *via* TMS.

**Results:**

A significant positive correlation was observed between Fugl-Meyer Assessment Wrist/Hand (FMA W/H) and MEP scores (*r* = 0.560, <0.001). Also, Fugl-Meyer Assessment Upper Extremity (FMA UE) scores demonstrated a moderate positive association with MEP responsiveness (*r* = 0.421, <0.001).

**Conclusion:**

MEP of the EDC muscle was found to be associated with sensorimotor control as measured by FMA. Moreover, FMA W/H score values might be a better prognostic indicator of EDC MEP responsiveness. Interestingly, a novel element comprising the range of FMA UE and FMA W/H components was observed to be a potential indicator of MEP responsiveness and could also indicate establishing FMA as a surrogate for TMS in resource-limited settings for prognostification.

## Introduction

In approximately one-third of the stroke survivor population, the upper extremity has been found to develop severe motor impairment, leading to a considerable impact on activities of daily living (ADL) (Prabhakaran et al., 2008; van Kuijk et al., 2009). It often leads to a severe deficit in 30–66% of patients, thereby impeding the recovery of motor function at 6 months post-stroke (Nath et al., 2022). In recent times, as the number of stroke incidences has increased substantially in the younger population, there is an urgent requirement to identify interventions to speed up upper limb recovery (Powell et al., 2019). Therefore, it is imperative to adopt a simplistic proactive approach based on the associativity of clinical parameters, especially in a densely populated, resource-limited country which might be useful in differentiating the patients with respect to motor outcomes and taking appropriate measures in clinical evaluations.

The Fugl-Meyer Motor Impairment Scale (FMA) (Fugl-Meyer et al., 1975) is an impairment-based cumulative numeric scoring system based on sequential post-stroke recovery stages, that is, from synergistic to voluntary movements (Hamaguchi et al., 2020). It has been established as a standardized measure to gauge upper extremity motor recovery. In addition, Transcranial Magnetic Stimulation (TMS) parameters also have been shown as a successful predictor of upper extremity motor restoration within 3 weeks of the onset of stroke as it can provide an objective evaluation of corticospinal tract integrity (CST) (Hendricks et al., 2002b; Potter-Baker et al., 2016).

Several studies have established the initial degree of paresis, the extent of injury to the corticospinal tract, and the pattern of recovery as clinical predictors of functional recovery incorporating various complex and advanced technological solutions (Hendricks et al., 2002a; Dodd et al., 2017). Furthermore, there has been an attempt to explore the potential of baseline motor evoked potential (MEP) in predicting functional recovery. Severe hand dysfunction post-stroke was correlated with potent inhibition of ipsilesional corticospinal excitability and a shift in excitability toward the contralesional hemisphere (Straudi et al., 2016; Schambra et al., 2019). Also, it was noted that patients with an absent MEP response had lower upper extremity motor scores at the beginning (Hendricks et al., 2003). Stinear et al. (2007) also found that MEP responsive group demonstrated substantial gains even after 36 months post-stroke, although the recovery potential of the upper limb declined over time (Stinear et al., 2007). Another study by Pizzi et al. (2009) described that patients with a baseline MEP response showed better functional recovery (as assessed by the Medical Research Council scale score and the Barthel Index scale) at 12 months post-stroke than patients without a baseline MEP response. Similarly, Lee et al. (2015), also showed that patients with baseline MEP response had better balance recovery than those without baseline MEP response after 4 weeks of the balance rehabilitation program (Lee et al., 2015).

Henceforth, the presence or absence of MEP post-stroke has been demonstrated as a reasonably good marker to predict the extent of motor recovery as it indicates the extent of corticospinal tract integrity in an individual patient. Also, the presence of MEP obtained by stimulating the ipsilesional cortex early in the post-stroke period is considered a potential sign of good recovery especially in the upper extremity as shown in Rapisarda et al. (1996); Hendricks et al. (2003), and Simis et al. (2016).

In the literature, the studies have proposed the initial degree of motor impairment and neurophysiological inputs such as motor threshold (MT) and the presence of MEP as the two most consistent and relevant predictors of upper-extremity motor recovery (Coupar et al., 2012). Taking into account the aforementioned literature, it has been observed that there is no accessible objective quantifiable evidence yet linking the cortical excitability as measured by MEP with the functional impairment of the upper extremity, especially the distal joints, that is, wrist and hand. Besides, there is a large variability in the studies as well in terms of population characteristics, clinical variables used, chronicity of stroke, and so on. In addition, clinical evaluation of sensorimotor function has been labeled as complex in presence of factors like cognitive incompetency. Moreover, the precise expression of neurophysiological factors at the neuronal level attenuating functional recovery is not clear (Singh et al., 2021b). Therefore, accurate straightforward neurophysiological evaluation and its association with the clinical parameters would strengthen the neurological assessment and determinateness of neural recovery.

Therefore, this study was conducted to employ an effective and simple approach to determine the relationship between FMA Upper Extremity (UE) scores, especially the FMA Wrist and Hand (W/H) scores, and the neurophysiological parameters, a potential tool of neuronal connection measurement and its association with cortico-muscular activity, to identify the patients with a potential for better motor recovery. Therefore, we aimed to determine any relation between FMA scores and TMS parameters, Resting Motor Threshold (RMT) and/or MEP, such that based on the outcome, in future, FMA might be used as a surrogate where TMS facility is practically not available.

## Materials and methods

### Patient enrolment

**FIGURE 1:**
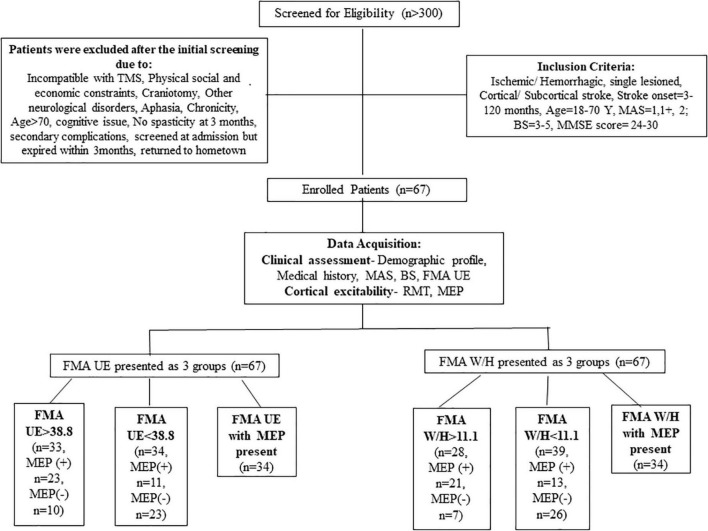
Patient enrolment and group consort.

More than 300 patients were screened in the outpatient clinic of the Department of Neurology, AIIMS, New Delhi, over 3 years from July 2016 to January 2019. A total of 67 patients (*n* = 67) were enrolled based on the inclusion criteria: age 18–70 years, having ischemic/hemorrhagic stroke within 3–120 months, Mini-Mental Scale = 24–30, Brunnstrom-stage (BS) = 3–5, Modified Ashworth Scale (MAS) = 1, 1+, 2. Patients demonstrating any contraindication to TMS and any other progressive neurological or cognitive disorders were excluded from the study (described in detail in Figure 1). Stroke was diagnosed clinically for all the enrolled patients by the neurologist. The Institutional Review Board, All India Institute of Medical Sciences (AIIMS), New Delhi, India, approved the study under protocol number IEC/NP-99/13.03.2015 and it was registered in the ISRCTN registry with clinical trial number ISRCTN95291802. All the patients signed the written informed consent before enrolment in the study.

### Demographic and clinical assessment

The clinical epidemiological information of patients (*n* = 67) enrolled includes the age, gender, history of Diabetes Mellitus (DM), Hypertension (HTN), Tobacco, Smoking, and alcoholism (Table 1). The average age of the patients was 45.67 ± 12.44 years and the average chronicity was 18.35 ± 22.65 months.

**TABLE 1:** Demographic and clinical characteristics of patients (*n* = 67).

		Participants (n)
Age (Mean ± SD)	45.67 ± 12.44 years	67
Age groups	18–30 years	7
	31–40 years	16
	41–50 years	20
	51–60 years	15
	61–70 years	9
Chronicity (Mean ± SD)	18.35 ± 22.65 months	
Gender	Male	54
	Female	13
Stroke type	Ischemic	50
	Hemmohhragic	17
Diabetes mellitus	Present	8
	Absent	59
Hypertension	Present	33
	Absent	34
Tobacco	Present	21
	Absent	46
Smoking	Present	21
	Absent	46
Alcohol consumption	Present	32
	Absent	35

### Evaluation of motor function and cortical excitability

All the patients underwent sensorimotor-control assessment between 3 and 120 months post-stroke onset by the FMA UE scale (0–66). FMA assessment was conducted by an experienced physiotherapist with a specialization in Neurology with more than 5 years of experience in handling patients with stroke. In this study, the potential of entire FMA UE scores covering aspects such as reflex activity, motor, and coordination/speed of UE impairment was explored while focusing on the motor component of the FMA W/H subscore (max 24) to establish its relationship with RMT and MEP of the Extensor Digitorum Communis (EDC) muscle for comprehensive evaluation. All the FMA assessments were conducted before the RMT and MEP acquisition session (using TMS) in a single day.

Cortico-spinal tract integrity was assessed, *via* stimulating the cortical representation area using the TMS (Magstim Rapid^2^, Magstim, United Kingdom). EDC, wrist and finger extensor, was selected as extensor muscles are often affected, weaker, and are easily detectable forearm muscle and it is imperative to achieve extensor action of wrist and fingers for accurate retraining of the hand function (Lazar, 1998; Koganemaru et al., 2020). The patients were seated comfortably, with the forearm in pronation, elbow in 90–120° flexion, wrist in the neutral position, and fingers at rest. The disposable gel-based wet Ag/AgCl electrodes were placed on the patient’s forearm in a bipolar configuration. Electrodes were then attached to the EMG amplifier connected with the TMS machine. RMT and MEP were acquired in a quiet room, and the patients were asked to relax fully a few minutes before the initiation of the investigation. The motor strip and adjacent areas of the ipsilesional hemisphere were located using the 10–20 EEG system and moving the coil in millimeters on the area in all directions (between Cz and C4 of the right primary motor cortex in left hemiparetic patients and between Cz and C3 of the left primary motor cortex in right hemiparetic patients with EEG cap worn before the start of the procedure). The area once identified was stimulated to determine whether an MEP is elicited. RMT was defined as the minimum stimulus intensity that elicited MEP in the relaxed target muscle and the peak-to-peak distance of this output signal is the MEP amplitude used in this study. The stimulator intensity was gradually raised if MEP could not be evoked and was increased to a maximum of 100% Maximum Stimulator Output (MSO) (Singh et al., 2021a). Thus, the presence of MEP was defined as the presence of at least five consecutive responses out of 10 attempts with peak-to-peak amplitude > 50 μV and these were averaged (Rossini et al., 2019; Singh et al., 2021a). In other words, if RMT was identified for the affected EDC muscle, the patient was classified as MEP-positive, MEP (+). If no RMT was identified for EDC muscle even after giving the MSO to the ipsilesional motor cortex, the patient was classified as MEP-negative, MEP (−). Also, the value of RMT was defined as 100% if no MEP could be evoked till 100% MSO (Lamola et al., 2016). Moreover, in our experience, as a result of stroke-related use-dependent plasticity in our cohort, the patient’s ipsilesional EDC hotspot was found to be located approximately little posterior, lateral or lateroposteriorly.

In the previous studies, the severity of paralysis as measured by FMA score was distributed as ≤25, 26–45, and 46–66 for severe, moderate, and mild paralysis, respectively (Gladstone et al., 2002; Woytowicz et al., 2017). Woytowicz et al. (2017) found that FMA baseline scores ≤ 34 are considered to be severe-moderate to the severe range. As the literature has no consensus built on the FMA severity range for segregating the patients, thus for our study, we allocated patients in groups based on the sample mean FMA UE scores and FMA W/H scores of our cohort (as shown in Figure 1).

### Data analysis

Statistical analysis was performed using IBM SPSS statistics version 26.0 (IBM Corp., Armonk, NY, United States). The normality of the data was assessed by the Q-Q plots and the Shapiro–Wilk test. Descriptive statistics for demographic and clinical parameters were undertaken. The mean of the FMA UE scores of the cohort was found to be 38.8. Similarly, the mean of the FMA W/H component of the cohort was observed as 11.1. A measure of the association like Pearson correlation (r) was undertaken to establish the relationship between FMA scores and TMS parameters (RMT and MEP). Furthermore, regression analysis was performed to identify the relationship trend among the FMA UE, FMA W/H, and TMS parameters. Therefore, it was of interest to evaluate any correlation between the patients with FMA UE > 38.8 and the MEP responsiveness compared to patients with FMA UE scores < 38.8. For this purpose, stratification of the main objectives was undertaken in the form of various sub-objectives as comparing entire FMA UE scores to MEP, FMA UE score > 38.8 to MEP (∼49% of our patient cohort) (Table 2), and FMA UE score < 38.8 to MEP (∼51% of our patient cohort) (Table 2) were conducted. Similarly, sub-objectives of comparison between the FMA W/H component and MEP were also undertaken (Figure 1). A *p* < 0.05 was considered statistically significant. Receiver Operating Characteristic (ROC) curve analysis was undertaken to assess the performance of FMA UE, FMA W/H, and MEP parameters in terms of sensitivity (Sn), specificity (Sp), and area under the curve (AUC) with a 95% confidence interval (CI) for identifying appropriate threshold values of FMA UE and FMA W/H. In two competing models, Model 1 being MEP amplitude predicted by a single factor: FMA scores alone; against a second Model 2 where MEP amplitude is predicted by the two factors: FMA score and their MEP status (MEP+ or MEP−) were evaluated by assessing the goodness of fit to explain the underlying data. The goodness of fit was assessed by measuring the adjusted *R*^2^ values; higher values of adjusted *R*^2^ indicate a better model fit.

**TABLE 2:** Frequency contingency table of FMA UE scores and RMT (+) or MEP (+).

	RMT (+) or MEP (+) (%)	RMT (−) or MEP (−) (%)	Total
FMA UE > 38.8	34.32	14.92	∼49
FMA UE < 38.8	16.41	34.32	∼51
Total	∼51	∼49	∼100

FMA UE (max 66), Fugl-Meyer Upper Extremity; MEP (+), Motor Evoked Potential present; MEP (−), Motor Evoked Potential absent; RMT (+), Resting Motor Threshold present; RMT (−), Resting Motor Threshold absent.

## Results

All the enrolled 67 patients (*n* = 67) with chronicity (18.35 ± 22.65 months) ranging from 3 to 120 months post-stroke and residual upper extremity deficits completed the study successfully. Among 3–120 months of chronicity, 14 patients were between 3 and 6 months, 42 patients were between 6 and 24 months, and 12 patients were between 24 and 120 months post-stroke. In this study, RMT and MEP acquisition for all patients was attempted with no side effects or adverse effects reported.

The mean FMA UE score of 38.8 for the complete sample was used to segregate the participants into two categories: Severe impairment falling in FMA UE < 38.8 (*n* = 34) and similarly mild impairment falling in FMA > 38.8 (*n* = 33). The mean FMA W/H score of the sample was found to be 11.1, it was also used in segregating the patients as severe impairment falling in FMA < 11.1 (*n* = 39) and, similarly, mild impairment falling in FMA > 11.1 (*n* = 28) (Table 1).

Statistical analysis revealed a significant correlation between the FMA W/H component and the RMT (*r* = 0.511, *p* < 0.001 (Supplementary Table 1), FMA W/H component and MEP responsiveness (*r* = 0.560, *p* < 0.001) (Table 3). Also, a positive association between the total FMA UE score and the RMT (*r* = 0.362, *p* = 0.002)(Supplementary Table 1), FMA UE score and MEP responsiveness was observed (*r* = 0.421, *p* < 0.001) (Table 3). As shown in Table 3 and Figure 1, out of the 67 patients, 34 patients had MEP present, MEP (+), and 33 did not have MEP, MEP (−).

**TABLE 3:** Relationship between FMA UE scores and FMA W/H scores with MEP.

	MEP (+)	MEP (−)	*R* [95% CI]	*R* ^2^	*p*-value	Mean ± SD FMA score
FMA UE SCORE	34	33	0.421 [0.20, 0.60]	0.177	**<0.001**	38.88 ± 9.08
FMA UE MEP (+)	34		0.383 [0.052, 0.64]	0.147	**0.025**	41.76 ± 9.74
FMA UE MEP (−)		33	*			35.9 ± 7.24
FMA UE > 38.8 (*n* = 33)	23	10	0.368 [0.028, 0.63]	0.135	**0.034**	
FMA UE < 38.8 (*n* = 34)	11	23	0.001 [−0.34, 0.34]	<0.001	0.993	
FMA W/H SCORE	34	33	0.560 [0.37, 0.71]	0.313	**<0.001**	11.17 ± 3.60
FMA W/H MEP (+)	34		0.470 [0.16, 0.70]	0.221	**0.004**	12.76 ± 3.81
FMA W/H MEP (−)		33	*			9.54 ± 2.47
FMA W/H > 11.1 (*n* = 28)	21	7	0.519 [0.18, 0.75]	0.269	**0.004**	
FMA W/H < 11.1 (*n* = 39)	13	26	0.063 [−0.26, 0.37]	0.004	0.701	

FMA UE (max 66), Fugl-Meyer Upper Extremity; FMA W/H (max 24), Wrist/Hand component of FMA; MEP (+), Motor Evoked Potential present; MEP (−), Motor Evoked Potential absent; R = correlation coefficient; R^2^, regression coefficient; *Can’t be determined.

### FMA UE and FMA W/H correlation with resting motor threshold

In some patients (*n* = 33), MEP was not evoked even till 100% MSO. Therefore, in those 33 patients where MEP was absent, RMT was taken as a value of 100%, as also suggested in the previous literature (Lamola et al., 2016). The linear regression analysis indicated that FMA UE (as predictive/independent variable) demonstrated a positive correlation with RMT value (as dependent variable) and can indicate RMT value in the entire patient cohort (*r* = 0.362, *F* = 9.80, *p* = 0.002) (Supplementary Figure 1A). Similarly, the linear regression analysis suggested a positive moderate correlation between the FMA W/H (as predictive/independent variable) with RMT value (as dependent variable) and indicate RMT value in the entire cohort (*r* = 0.511, *F* = 23.03, *p* < 0.001) (Supplementary Figure 2A). Besides that, only FMA W/H > 11.1 (as predictive/independent variable) with RMT value (as dependent variable) demonstrated moderate associativity (*r* = 0.557, *F* = 11.71, *p* = 0.002) (Supplementary Figure 2B). However, if we consider FMA UE scores > 38.8 (*r* = 0.287, *F* = 2.80, *p* = 0.104) (Supplementary Figure 1B), FMA UE scores < 38.8 (*r* = 0.077, *F* = 0.19, *p* = 0.661) (Supplementary Figure 1C), and FMA W/H < 11.1 (*r* = 0.025, *F* = 0.02, *p* = 0.876) (Supplementary Figure 2C), it did not demonstrate significant relationship with RMT value (as a dependent variable). Similarly, for the RMT present, RMT (+) group, the association between FMA UE scores with RMT value (*r* = 0.222, *F* = 1.66, *p* = 0.206) (Supplementary Figure 1D) and FMA W/H scores with RMT value (*r* = 0.335, *F* = 4.05, *p* = 0.052) (Supplementary Figure 2D) were found to be non-significant (as described in Supplementary Table 1). Therefore, further analysis was carried out to evaluate if any correlations exist between the FMA scores and MEP as described below.

### FMA UE correlation with the motor evoked potential

**FIGURE 2:**
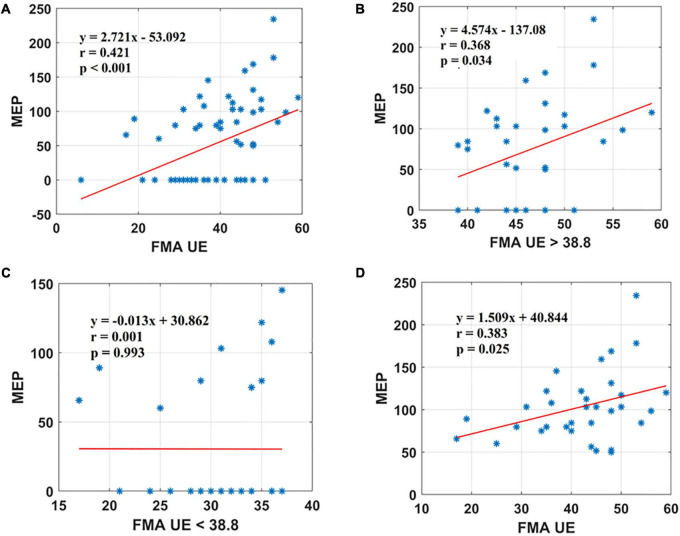
Scatter plot showing the relationship between the **(A)** FMA UE scores and the MEP amplitude values for all the patients, **(B)** FMA UE scores > 38.8 and the MEP amplitude values obtained, **(C)** FMA UE < 38.8 and the MEP amplitude values, **(D)** FMA UE scores and the MEP amplitude values for all patients in MEP (+) group. *Represents the data of each patient.

Motor evoked potential of the EDC muscle as the cortical excitability parameters from TMS measures denoted a relationship with the sensorimotor control as measured by FMA UE scores. The first parameter, FMA UE scores, was found to be significantly associated with the MEP responsiveness in the ipsilesional hemisphere. The linear regression analysis indicated that FMA UE (as predictive/independent variable) is positively correlated with MEP (as dependent variable) and can indicate MEP in the entire cohort (*r* = 0.421, *F* = 14.04, *p* < 0.001) (Figure 2A). This correlation was further explored in the sub-objectives for comprehensive evaluation. First, the sub-objectives reported the relationship between FMA scores above the sample mean (>38.8) and MEP obtained (Table 3 and Figure 1). Among 67 patients, 33 patients had FMA scores > 38.8; of these 33 patients, MEP was evoked in only 23 patients (Table 3 and Figure 1). The linear regression analysis suggested a positive correlation between FMA UE scores > 38.8 (as predictive/independent-variable) with MEP (as dependent variable) (*r* = 0.368, *F* = 5.87, *p* = 0.034) (Figure 2B). This correlation was found to be insignificant in FMA UE scores < 38.8 and MEP (*r* = 0.001, *F* < 0.001, *p* = 0.993) (Table 3 and Figure 2C). However, if we consider only MEP (+) group (Figure 1), a significantly positive correlation is demonstrated between FMA UE scores with MEP (*r* = 0.383, *F* = 5.53, *p* = 0.025) (Figure 2D).

### FMA W/H correlation with the motor evoked potential

**FIGURE 3:**
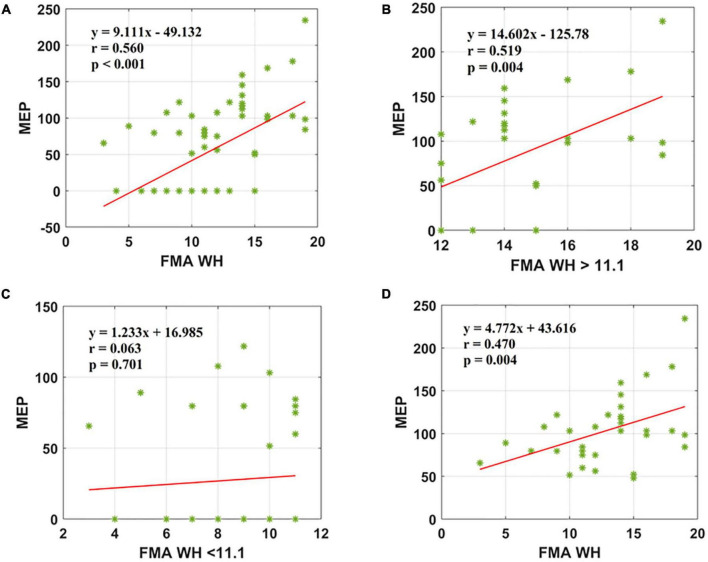
Scatter plot showing the relationship between the **(A)** FMA W/H scores and the MEP amplitude values for all the patients, **(B)** FMA W/H scores > 11.1 and the MEP amplitude values obtained, **(C)** FMA W/H scores < 11.1 and the MEP amplitude values, **(D)** FMA W/H scores and the MEP amplitude values for all patients in MEP (+) group. *Represents the data of each patient.

The second parameter, the FMA W/H scores, was found to establish a significant association with MEP responsiveness in the ipsilesional hemisphere. The linear regression model indicated that FMA W/H (as predictive/independent variable) exhibited a moderate positive correlation with MEP (as dependent variable) and indicate MEP of the entire cohort (*r* = 0.560, *F* = 29.71, *p* < 0.001) (Figure 3A). Considering the sub-objectives as described earlier, the relationship between FMA W/H scores above the sample mean (>11.1) and MEP was obtained (Table 3 and Figure 1). Among 67 patients, 28 patients had FMA W/H scores > 11.1. Out of these 28 patients, MEP was evoked in 21 patients (Table 3 and Figure 1). Here, the linear regression analysis suggested a moderate positive correlation between FMA W/H scores > 11.1 (as predictive/independent-variable) with MEP (as dependent variable) (*r* = 0.519, *F* = 9.61, *p* = 0.004) (Figure 3B). This correlation was found to be insignificant in FMA W/H scores < 11.1 and MEP (*r* = 0.063, *F* = 0.148, *p* = 0.701) (Table 3 and Figure 3C). However, if only MEP (+) group was considered (Figure 1), a significantly moderate positive correlation is demonstrated between FMA W/H scores with MEP (*r* = 0.470, *F* = 9.10, *p* = 0.004) (Figure 3D).

### Receiver operating characteristic analysis for identifying the motor evoked potential responders

**FIGURE 4:**
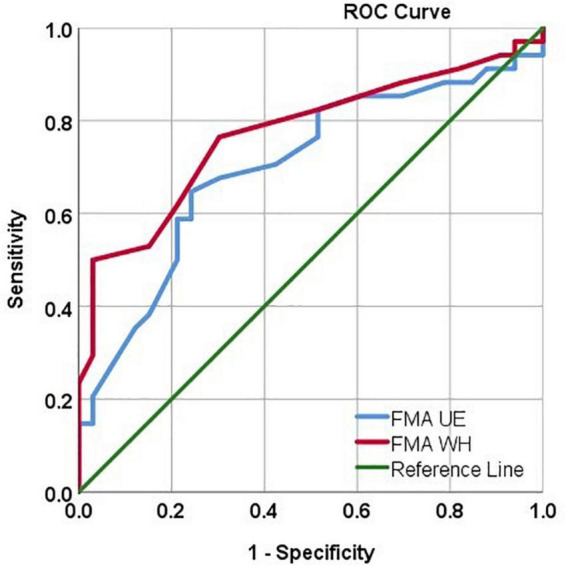
ROC curve analysis depicting the FMA UE and FMAW/H values in identifying MEP responders. FMA UE produced AUC = 0.70, sensitivity = 67.6%, specificity = 69.7% in identifying MEP responders. FMA W/H produced AUC = 0.77, sensitivity = 76.5%, specificity = 69.7% in identifying MEP responders.

Using the ROC analysis, FMA UE showed Sn = 67.6%, Sp = 69.7%, and AUC = 0.70 [CI: 0.57, 0.82] at a threshold ≥ 38. Similarly, FMA W/H showed Sn = 76.5%, Sp = 69.7%, and AUC = 0.77 [CI: 0.65, 0.88] at a threshold ≥ 10.5 in identifying the MEP responders (Figure 4).

### Contribution of FMA scores and MEP status (MEP+ or MEP−) to motor evoked potential amplitude

To determine the contribution of FMA scores and MEP status (MEP+ or MEP−) to MEP amplitude, we used the comparison of adjusted *R*^2^ value analysis. The MEP amplitude was indicated by FMA UE scores (*R*^2^ = 0.177, adjusted *R*^2^ = 0.165, *p* < 0.001) and MEP status (*R*^2^ = 0.804, adjusted *R*^2^ = 0.798, *p* < 0.001). Similarly, MEP amplitude was indicated by FMA W/H scores (*R*^2^ = 0.313, adjusted *R*^2^ = 0.303, *p* < 0.001) and MEP status (*R*^2^ = 0.817, adjusted *R*^2^ = 0.811, *p* < 0.001). This analysis indicated that adding MEP status (as model 2) to the MEP amplitude and FMA scores (FMA UE and FMA W/H scores) (as model 1) was a better model for estimating the MEP amplitude.

## Discussion

This study included sub-acute and chronic patients to explore the associativity between clinical impairment (FMA) and the neurophysiological parameters (RMT and MEP). In this study, the patients were segregated based on the sample mean scores of FMA UE and FMA W/H, which resulted in MEP (+) (*n* = 34) and MEP (–) (*n* = 33) groups (Table 3 and Figure 1). To maintain consistences in the MEP acquisition in the patient cohort, we used the upper limit of 100% MSO. It was observed that ∼49% of data were >38.8 (mean FMA) (Table 2) and ∼51% of the data were <38.8 (mean FMA) (Table 2), and similarly, ∼51% of data showed MEP response and ∼49% of data did not show MEP response, that is, MEP (–). In addition, in FMA scores > 38.8 group, 14.92% of our patient cohort (*n* = 67) did not report MEP, and in FMA scores < 38.8 group, 16.41% of our patient cohort (*n* = 67) reported MEP. The FMA UE scores showed the associativity with MEP in terms that when FMA UE > 38.8 (sample mean ∼38.8), the MEP responsiveness is high (MEP+), and FMA UE < 38.8, the MEP responsiveness is less, that is, absence of MEP. However, as flexor hypertonia of the hand is considered one of the most debilitating representations of post-stroke spasticity and the early initiation of the extension activity in the wrist and fingers, by the EDC muscle, it is indexed as a sign of functional recovery (Wolf et al., 2006; Kuo and Hu, 2018). In accordance with the literature, here FMA W/H component was found to be considered a better indicator of the corticospinal tract integrity for the distal upper extremity as measured by the RMT and MEP of the EDC muscle.

This study demonstrated a reasonably good relationship between the clinical and neurophysiological parameters in patients with sub-acute and chronic stroke. The sensorimotor control as measured by the FMA UE scale established a positive linear correlation with the MEP responsiveness.

### FMA UE and FMA W/H with resting motor threshold

The FMA UE scores showed a trend toward a positive correlation with the RMT (*r* = 0.362 [CI: 0.13, 0.55]) (Supplementary Table 1 and Supplementary Figure 1A). Supplementary Figure 2A represents the significant linear correlation for FMA W/H score with RMT for all the patients from the cohort (*r* = 0.511 [CI: 0.31, 0.67]). It is noteworthy that the correlation is slightly higher with FMA W/H and with narrow and better CI than the FMA UE.

### FMA UE with motor evoked potential

The FMA UE scores showed a trend toward a positive, moderate correlation with the MEP (Figure 2A). In Figure 2B, 23 out of 33 patients with FMA UE scores yielded an MEP response. Similarly, in Figure 2C, 23 patients out of 34 with FMA W/H scores did not yield an MEP response. Figure 2D represents the linear correlation for FMA UE score with MEP amplitude for all the patients from cohort MEP (+). Several studies in the literature have assessed the relationship between FMA scores and MEP in terms of prognostic indicators for recovery. van Kuijk et al. (2009) have demonstrated that registered MEP has the same predictive value as clinical assessment of upper extremity motor deficit measured by the FMA UE scale at 3 weeks post-stroke.

### FMA W/H with motor evoked potential

Subsequently, the same experiment was conducted for the entire cohort with the FMA W/H scores, and a strong positive correlation with the MEP was found to be significant (Figure 3A). However, as elaborated in the results section, among 34 patients with MEP present, 21 patients had mean FMA W/H scores > 11.1 and 13 patients had mean FMA W/H scores < 11.1. This correlation is in contrast to other studies, where the Abductor Digiti Minimi (ADM) muscle was found to be a significant predictor of functional recovery (Hendricks et al., 2003). In Figure 3B, 21 out of 28 patients with FMA W/H scores yielded an MEP response. Similarly, in Figure 3C, 26 out of 34 patients with FMA W/H scores did not yield an MEP response.

It is worth noting that these findings are in accordance with the study by van Kuijk et al. (2009), as they also noticed that the corticospinal tract integrity was highly relatable to normal hand function rather than proximal upper limb function. In a study by Schambra et al., no difference was noted in FMA-UE score recovery with the presence or absence of MEP in the acute phase, however, the study noted less improvement in patients with high FMA scores than with low FMA scores. In addition, it was also observed that FMA recovery curves plateaued below the reported literature normal levels for both arm and hand (Schambra et al., 2019).

Inconclusive evidence from the literature inspired us to perform in-depth analysis in this study and it was found that FMA UE scores showed a positive correlation in the MEP (+) group (Table 3 and Figure 2D). Also, FMA W/H scores were in inclination toward positive, moderate correlation in the MEP (+) group (Table 3 and Figure 3D). The FMA UE and FMA W/H correlation with MEP was found to be significant at *p* < 0.05 and *p* < 0.01, respectively. Consequently, the assumption of segregating the patients on the sample mean scores of FMA UE and FMA W/H were found to be in inclination with the ROC analysis which suggested that FMA UE threshold ∼38 and FMA W/H threshold ∼11 were found to be ∼67 and ∼76% sensitive and ∼69 and ∼69% specific for indicating MEP responsiveness, respectively. This study also incorporated a comparison of adjusted *R*^2^ values to assess the goodness of fit of two competing models. First, considering FMA UE scores and MEP amplitude in model 1, variable FMA UE scores alone yielded results as *R*^2^ = 0.177, adjusted *R*^2^ = 0.165, *p* < 0.001, while adding the MEP status (MEP+ or MEP−) as model 2 resulted in *R*^2^ = 0.804, adjusted *R*^2^ = 0.798, *p* < 0.001. Second, considering FMA W/H scores and MEP amplitude in model 1, variable FMA W/H scores alone yielded results as *R*^2^ = 0.313, adjusted *R*^2^ = 0.303, *p* < 0.001, while adding the MEP status (MEP+ or MEP−) as model 2 resulted in *R*^2^ = 0.817, adjusted *R*^2^ = 0.811, *p* < 0.001. Hence, as observed from the analysis, the addition of MEP status to the clinical FMA scores (FMA UE and FMA W/H) enhances the estimation capability of the model for the MEP amplitude. On further evaluation, the mean of the FMA UE and FMA W/H for the MEP (+) group was found to be 41.76 and 12.76 and for the MEP (−) group was found to be 35.9 and 9.54, respectively, whereas the entire cohort means were 38.8 and 11.1, respectively.

Hence, we have tried to present an objective range of the FMA UE scores (35.9–41.7) and FMA W/H scores (9.54–12.7) that can be used to potentially identify the patient cohort and might potentially indicate the MEP responsiveness and could also probably serve as a surrogate for TMS in resource-limited settings. From the pathophysiological perspective, contrary to proximal muscles, the motor neurons innervating the intrinsic muscles of the hand involved in fine manipulation activities and finger movements receive direct, monosynaptic inputs from the corticospinal tract (Siebner and Rothwell, 2003; Brouwer and Schryburt-Brown, 2006). Therefore, corticospinal tract integrity is considered a prerequisite for normal hand function than the complete arm function. There is still a limitation in addressing the range of impairment scores that can differentiate the most and least potential candidates for neuroplastic recovery. Therefore, it was found imperative to highlight this novel perspective in this new dimension, thereby, making it concise and objective to understand and plan the rehabilitation strategies according to the target patient populations. Furthermore, unlike the previous strategies incorporated in literature, we have tried to illustrate a simplistic approach to the objective neurological assessment in a resource-limited setting, in a country like India. It will enable the bedside/Out-patient clinic procedures to be conducted instead of sophisticated investigations, such as MRI, Electromyography (EMG), and invasive techniques such as biomarkers like Vascular Endothelial Growth Factor (VEGF) biomarker and Brain-Derived Neurotrophic Factor (BDNF) biomarker, by using the mean of FMA UE and FMA W/H scores of the cohort for segregating the sample and anticipate the MEP responsiveness status as prognostic indicator.

### Limitations and future directions

There were a few limitations of this study. First, only the clinical and neurophysiological parameters for assessment like RMT and MEP were incorporated, but in the literature, it has been documented that motor function recovery may also be because of recruitment of other pathways other than the corticospinal tract during cortical reorganization (Ward et al., 2006). Although MEP does provide useful information about neuronal membrane excitability and functional and conductional integrity of CST, however, other forms of assessment like F-wave, H-wave, and adding neuroimaging studies along with clinical and TMS evaluation could also be explored in the future for better understanding (Crafton et al., 2003). Second, the sample size included in our study was relatively low; despite that, it is worth considering as the study led to novel findings in the sensorimotor assessment. In addition, this study also reported the CI of the correlation coefficients in a wide range, even with a moderate correlation coefficient and *p* < 0.05, which indicates a high heterogeneity in the small cohort; this might be explored further appropriately in future studies with a larger cohort. Furthermore, the intensity of the stimuli was set at 100% of MSO; therefore, the findings could not be extrapolated and generalized to other TMS intensities. The present study also suffers from the risk of false-negative interpretations of MEP in the cohort due to large variability; in the future, large cohort studies with advanced modeling will be highly beneficial in finding the potential threshold of FMA UE and FMA W/H scores that can serve as a more robust prognostic indicator of cortical excitability.

## Conclusion

The study is an attempt to estimate the range of the FMA UE scores and FMA W/H scores to possibly indicate ipsilesional corticospinal tract excitability by TMS induced-MEP. This associativity between sensorimotor impairment and neurophysiological parameters might be of clinical relevance in comprehensive and concise disease diagnosis and prognosis. It could also serve as a potential tool for clinically identifying a pool of patients in the wide spectrum of stroke recovery and also to highlight the importance of FMA as a surrogate to TMS in resource-limited settings. Furthermore, it can be carried forward with a wide array of diagnostic tools, therapeutic strategies, and other populations of neurological disorders.

## Data availability statement

The raw data supporting the conclusions of this article will be made available by the authors, without undue reservation.

## Ethics statement

The studies involving human participants were reviewed and approved by the Institutional Review Board (IRB), All India Institute of Medical Sciences (AIIMS), New Delhi, India. The patients/participants provided their written informed consent to participate in this study.

## Author contributions

MS and AM conceptualized and designed the study. AM led the study and provided the scientific inputs. MS performed a literature survey, patient recruitment, data collection, data analysis, data interpretation, and wrote the manuscript. NS provided the scientific inputs, data analysis, and interpretation. NK and MVPS provided the scientific inputs, clinical support, and clinical resources for experiments. AM reviewed the manuscript at multiple iterations with MS. All the authors read and approved the ultimate version of the manuscript.
