# Supplementary material for: A novel perspective of associativity of upper limb motor impairment and cortical excitability in sub-acute and chronic stroke
Source: Front Neurosci. 2022 Jul 25;16:832121. doi: 10.3389/fnins.2022.832121 (PMC9358254; doi:10.3389/fnins.2022.832121)
Supplement: Supplementary file 1 [file Data_Sheet_1.PDF]

# Supplementary Material

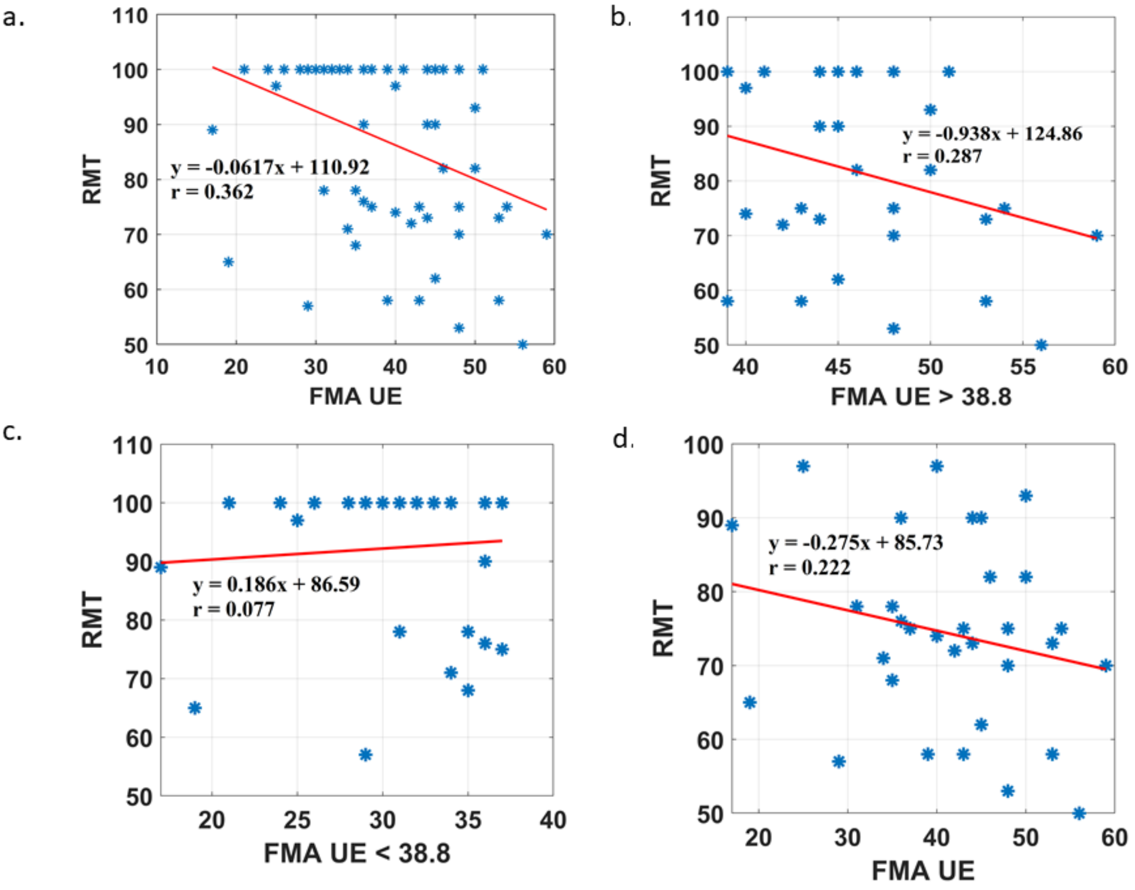


**Supplement Fig 1:** Scatter plot showing the relationship between the **a** FMA UE scores and the RMT values for all the patients, **b** FMA UE scores>38.8 and the RMT values obtained, **c** FMA UE<38.8 and the RMT values, **d** FMA UE scores and the RMT values for all patients in RMT (+) group.

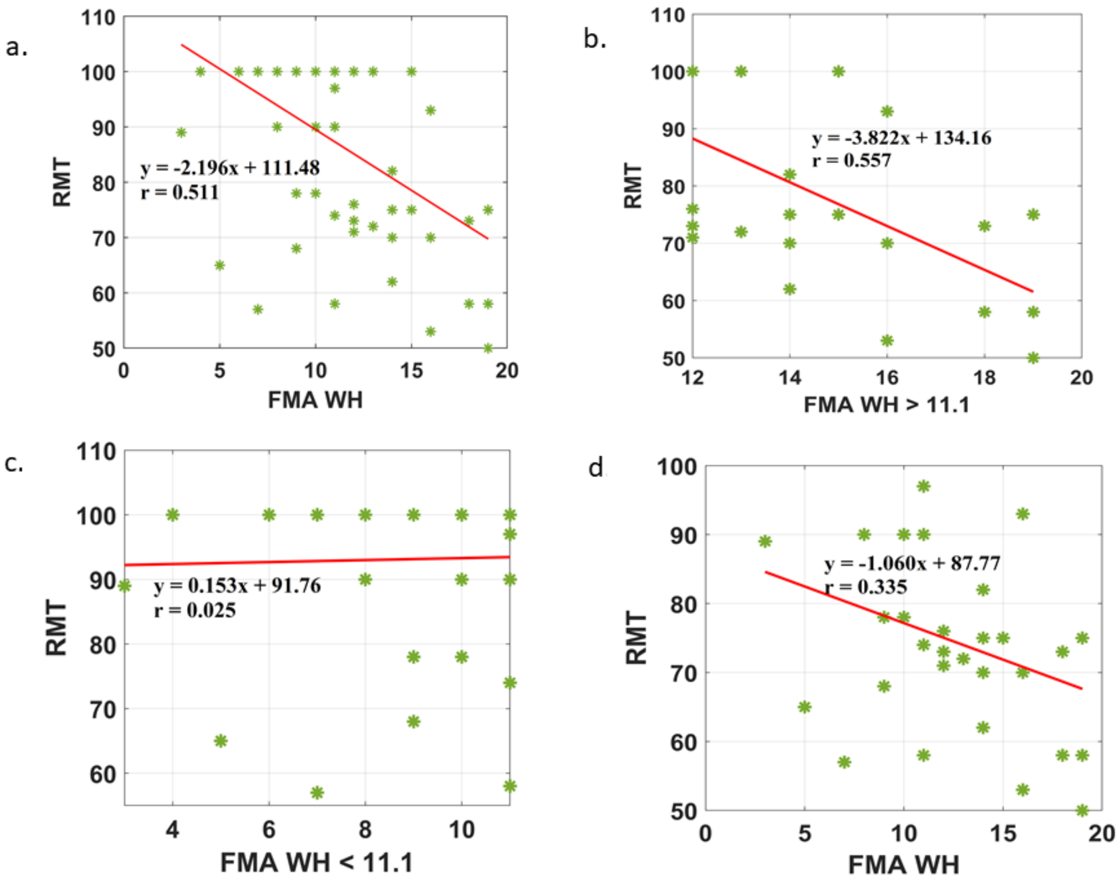


**Supplement Fig 2:** Scatter plot showing the relationship between the **a** FMA W/H scores and the RMT values for all the patients, **b** FMA W/H scores>11.1 and the RMT values obtained, **c** FMA W/H scores<11.1 and the RMT values, **d** FMA W/H scores and the RMT values for all patients in RMT (+) group.

**Supplement Table 1:** Relationship between FMA UE scores and FMA W/H scores with RMT

| | RMT (+) | RMT (-) | **R** [95% CI] | $R^2$ | p value | Mean±SD FMA score |
|---|---|---|---|---|---|---|
| **FMA UE SCORE** | 34 | 33 | 0.362 [0.13,0.55] | 0.131 | **0.002** | 38.88±9.08 |
| **FMA UE RMT (+)** | 34 | | 0.222 [-0.13,0.52] | 0.049 | 0.206 | 41.76±9.74 |
| **FMA UE RMT (-)** | | 33 | * | | | 35.9±7.24 |
| **FMA UE>38.8(n=33)** | 23 | 10 | 0.287 [-0.062,0.57] | 0.082 | 0.104 | |
| **FMA UE<38.8(n=34)** | 11 | 23 | 0.077 [-0.27,0.4] | 0.006 | 0.661 | |
| | | | | | | |
| **FMA W/H SCORE** | 34 | 33 | 0.511 [0.31, 0.67] | 0.261 | **<0.001** | 11.17±3.60 |
| **FMA W/H RMT (+)** | 34 | | 0.335 [-0.0036, 0.6] | 0.112 | 0.052 | 12.76±3.81 |
| **FMA W/H RMT (-)** | | 33 | * | | | 9.54±2.47 |
| **FMA W/H>11.1 (n=28)** | 21 | 7 | 0.557 [0.23, 0.77] | 0.310 | **0.002** | |
| **FMA W/H<11.1 (n=39)** | 13 | 26 | 0.025 [-0.29, 0.34] | <0.001 | 0.876 | |

*FMA UE (max 66): Fugl-Meyer Upper Extremity, FMA W/H (max 24): Wrist / Hand component of FMA, RMT (+): Resting Motor Threshold present, RMT (-): Resting Motor Threshold absent, R=correlation coefficient,* $R^2$*: regression coefficient, *: Can't be determined*
